# Cyclic deformation and fatigue data for Ti–6Al–4V ELI under variable amplitude loading

**DOI:** 10.1016/j.dib.2017.05.032

**Published:** 2017-05-19

**Authors:** Patricio E. Carrion, Nima Shamsaei, Robert D. Moser

**Affiliations:** a Laboratory for Fatigue & Additive Manufacturing Excellence (FAME), Department of Mechanical Engineering, Auburn University, Auburn, AL 36849, USA; b US Army Engineer Research and Development Center, Vicksburg, MS 39180, USA

**Keywords:** Cyclic deformation, Fatigue, Strain-life, Titanium, Block-loading, Periodic overload

## Abstract

This article presents the strain-based experimental data for Ti–6Al–4V ELI under non-constant amplitude cyclic loading. Uniaxial strain-controlled fatigue experiments were conducted under three different loading conditions, including two-level block loading (i.e. high-low and low-high), periodic overload, and variable amplitude loading. Tests were performed under fully-reversed, and mean strain/stress conditions. For each test conducted, two sets of data were collected; the cyclic stress–strain response (i.e. hysteresis loops) in log_10_ increments, and the peak and valley values of stress and strain for each cycle. Residual fatigue lives are reported for tests with two-level block loading, while for periodic overload and variable amplitude experiments, fatigue lives are reported in terms of number of blocks to failure.

**Specifications Table**

**Table:** 

Subject area	Engineering
More specific subject area	Fatigue of Metals
Type of data	Table (Microsoft Excel file format)
How data was acquired	Strain-controlled fatigue experiments (laboratory)
Data format	Raw and analyzed
Experimental factors	The material used was mill-annealed wrought Ti–6Al–4V ELI bar, manufactured in compliance with ASTM standard F136-13 [1]. Cylindrical fatigue specimens with uniform gage section were designed following ASTM standard E606/E606M-12 [2]. The specimens were polished to achieve 0.5 μm surface finish in the gage section. Three coats of acrylic M-coat D were applied on the gage section to protect the specimens surface from the extensometer blades during testing.
Experimental features	Strain-controlled fatigue experiments were conducted following ASTM E606/E606M-12 [2]. All fatigue tests were conducted at room temperature (~23 °C), and 38% relative humidity. The applied test frequencies were adjusted to minimize any strain rate effects on the test results.
Data source location	Center for Advanced Vehicular Systems (CAVS), Mississippi State University, MS, USA
Data accessibility	https://dataverse.harvard.edu/dataset.xhtml?persistentId=doi:10.7910/DVN/SUCU5X

**Value of the data**

• Fatigue damage in most applications is commonly caused by variable and complex loadings. In some applications such as aerospace and biomedical, where Ti–6Al–4V ELI has been widely used as a structural material, understanding the fatigue behavior of the material is extremely important since majority of failures in structural components are attributed to fatigue damage.
• The presented data offers a representation of Ti–6Al–4V ELI mechanical behavior in a controlled environment, thus contributing to the fundamental knowledge about this structural material. The data is also valuable as a baseline for other special applications (i.e. additive manufactured medical implants and aerospace components), or to compare with newly developed/improved materials.
• The data presented in this article can be used for fatigue behavior and cyclic deformation related research on Ti–6Al–4V ELI under more realistic loading conditions. This data can be used to develop/calibrate constitutive models, cumulative fatigue damage models, and cycle counting methods.

## 1 Data

Strain-controlled block loading (i.e. high-low (H-L), low-high (L-H)), periodic overloading (PO), and variable amplitude (VA) fatigue data of Ti–6Al–4V ELI (a titanium alloy) is presented in this article. H-L, L-H, and PO experiments were conducted using fully-reversed (*R*_*ε*_ =−1), and pulsating (*R*_*ε*_ = 0) strain loadings with various strain amplitudes, *ε*_*a*_. The strain ratio, *R*_*ε*_, is defined as *R*_*ε*_ = *ε*_*min*_/*ε*_*max*_ and strain amplitude, *ε*_*a*_, is defined as *ε*_*a*_ = (*ε*_*max*_−*ε*_*min*_)/2, where *ε*_*min*_ is the minimum strain and *ε*_*max*_ is the maximum strain. The VA tests utilized a variable amplitude loading spectrum (i.e. load history) of various strain amplitudes, *ε*_*a*_, and strain ratios, *R*_*ε*_. For each test condition, two types of data were recorded. These include the cyclic (i.e. hysteresis loops) stress–strain responses recorded in log_10_ increments, and the maximum (peak) and minimum (valley) values of stress and strain for each cycle. All relevant data has been made available in the Data in Brief (DiB) Dataverse:

https://dataverse.harvard.edu/dataset.xhtml?persistentId=doi:10.7910/DVN/SUCU5X.

## 2 Experimental design, materials and methods

**Fig. 1:**
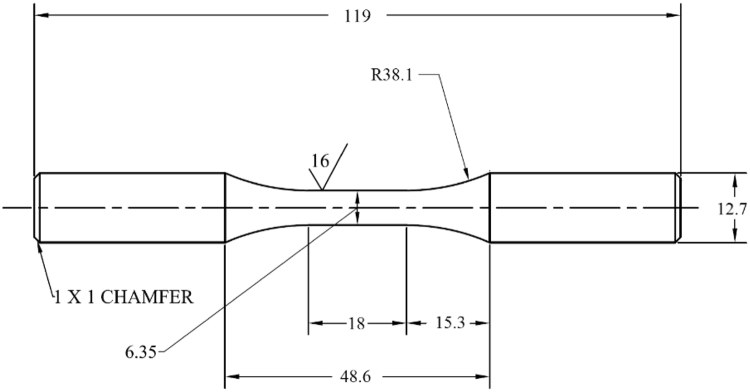
Geometry and dimensions of round fatigue specimens with uniform gage section per ASTM standard E606/E606M-12 [2], [3]. All dimensions are presented in mm.

Round fatigue specimens with uniform gage section and the dimensions and geometry, shown in Fig. 1, were designed according to ASTM standard E606/E606M-12 [2]. The specimens were fabricated from 12.7 mm diameter round bars of Ti–6Al–4V ELI, received in a mill-annealed condition (annealed for 1 h at 1300 °F). All fatigue experiments were conducted under strain-controlled condition at room temperature, utilizing an MTS extensometer model 634.31F-25, a servohydraulic test frame with 100 kN load cell capacity, and a sinusoidal waveform input. Tests were performed using strain amplitudes that ranged from 0.0015 to 0.012 mm/mm. Influence of strain rate effects was minimized by adjusting the test frequency, ranging from 0.5 to 5 Hz, for each test condition to maintain a relatively consistent average strain rate for all tests. In addition, for each prescribed test condition, duplicate tests were conducted to validate the collected data and ensure the repeatability of experiments. Further details on the experimental program are presented in the following subsections according to the type of loading utilized.

### 2.1 High-low (H-L) and low-high (L-H) block loading

**Fig. 2:**
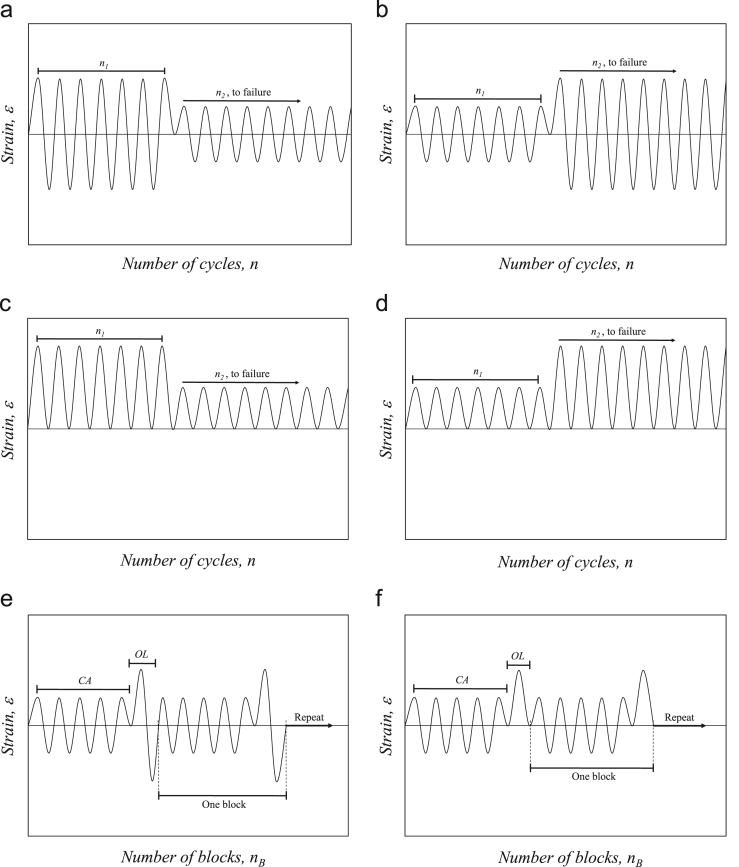
Schematic of loading sequences used for block loading, including (a) H-L with *R*_*ε*_=−1, (b) L-H with *R*_*ε*_=−1, (c) H-L with *R*_*ε*_ =0, (d) L-H with *R*_*ε*_ =0, (e) PO with *R*_*ε*_ =−1, and (f) PO with *R*_*ε*_ =−1/*R*_*ε*_=0.

Constant strain amplitude (*CA*) fatigue tests were conducted using high-low (H-L) or low-high (L-H) block loading with various combinations of strain amplitudes, *ε*_*a*_, under fully-reversed (*R*_*ε*_ =−1) and pulsating-tension (*R*_*ε*_ = 0) conditions. Fig. 2(a) and (b) displays schematics of the two-level block loading using *R*_*ε*_ =−1, where *n*_*1*_ represents the number of cycles for the first loading block and *n*_*2*_ denotes the number of cycles for the second loading block until the failure of specimens. Fig. 2(c), and (d) illustrates a schematic of the loading using *R*_*ε*_ =0. Table 1 provides a summary of the strain-controlled H-L and L-H fatigue tests. The table includes the specimen ID, loading sequence (H-L or L-H), frequency of the first and second loading blocks, *f*_*1*_ and *f*_*2*_, strain amplitude for the first and second loading blocks, *ε*_*a1*_ and *ε*_*a2*_, and the number of cycles for first and second loading blocks, *n*_*1*_ and *n*_*2*_.

**Table 1:** Summary of high-low (H-L) and low-high (L-H) fatigue tests for Ti–6Al–4V ELI.

**Specimen ID**	**Loading sequence**	***f***_***1***_	***f***_***2***_	***ε***_***a1***_	***ε***_***a2***_	***n***_***1***_	***n***_***2***_
		**Hz**	**Hz**	**mm/mm**	**mm/mm**	**cycles**	**cycles**
**Fully-reversed,*****R***_***ε***_=**−1**							
H-L_0.012-0.006(1)	H-L	0.5	3.0	0.012	0.006	642	13,681
H-L_0.012-0.006(2)	H-L	0.5	3.0	0.012	0.006	642	14,241
H-L_0.010-0.006(1)	H-L	1.0	3.0	0.010	0.006	1024	23,695
H-L_0.010-0.006(2)	H-L	1.0	3.0	0.010	0.006	1024	25,443
H-L_0.010-0.005(1)D0.25	H-L	1.0	5.0	0.010	0.005	512	37,063
H-L_0.010-0.005(3)D0.25	H-L	1.0	5.0	0.010	0.005	512	35,686
H-L_0.010-0.005(1)D0.50	H-L	1.0	5.0	0.010	0.005	1024	24,114
H-L_0.010-0.005(2)D0.50	H-L	1.0	5.0	0.010	0.005	1024	22,180
H-L_0.010-0.005(1)D0.75	H-L	1.0	5.0	0.010	0.005	1536	23,914
H-L_0.010-0.005(2)D0.75	H-L	1.0	5.0	0.010	0.005	1536	18,928
H-L_0.008-0.006(3)	H-L	1.0	3.0	0.008	0.006	3407	45,021
H-L_0.008-0.006(4)	H-L	1.0	3.0	0.008	0.006	3407	59,475
H-L_0.007-0.006(3)	H-L	2.0	3.0	0.007	0.006	12,373	32,384
L-H_0.006-0.010(1)	L-H	3.0	1.0	0.006	0.010	38,384	2823
L-H_0.006-0.010(2)	L-H	3.0	1.0	0.006	0.010	38,384	2161
L-H_0.006-0.008(1)	L-H	3.0	1.0	0.006	0.008	38,384	21,307
L-H_0.006-0.008(2)	L-H	3.0	1.0	0.006	0.008	38,384	12,245
L-H_0.005-0.010(1)	L-H	5.0	1.0	0.005	0.010	500,000	3209
L-H_0.005-0.010(2)	L-H	5.0	1.0	0.005	0.010	500,000	2316

**Mean strain,*****R***_***ε***_=**0**							
H-L_0.010-0.006(1)R0	H-L	0.5	1.0	0.010	0.006	1524	29,261
H-L_0.010-0.006(2)R0	H-L	0.5	1.0	0.010	0.006	1524	33,972
H-L_0.008-0.006(1)R0	H-L	0.5	1.0	0.008	0.006	3175	37,359
H-L_0.008-0.006(2)R0	H-L	0.5	1.0	0.008	0.006	3175	32,793
L-H_0.006-0.010(1)R0	L-H	1.0	0.5	0.006	0.010	15,345	1666
L-H_0.006-0.010(2)R0	L-H	1.0	0.5	0.006	0.010	15,345	2906
L-H_0.006-0.008(1)R0	L-H	1.0	0.5	0.006	0.008	15,345	5605
L-H_0.006-0.008(2)R0	L-H	1.0	0.5	0.006	0.008	15,345	9336

### 2.2 Periodic overloading (PO)

Periodic overloading (PO) fatigue tests were conducted using a loading block with a predetermined number of cycles under selected constant strain amplitude, followed by 1 cycle of overloading. The PO experiments were performed using various combinations of strain amplitudes, *ε*_*a*_, under *R*_*ε*_ =−1 and 0 conditions. Fig. 2(e) illustrates the schematic of the first loading combination, where the *CA* with *R*_*ε*_ =−1 was applied for 100 cycles, followed by an overload strain amplitude, *OL*, with *R*_*ε*_ =−1 for 1 cycle. The loading block was repeatedly applied until the specimen reached failure. Fig. 2(f) shows the schematic of the second loading combination, where the constant strain amplitude, *CA*, with *R*_*ε*_ =−1 was applied for 100 cycles, followed by an overload, *OL*, with *R*_*ε*_ =0 for 1 cycle. The collected data from strain-controlled PO fatigue tests are tabulated in Table 2. The experimental details presented in Table 2 include the specimen ID, the overload and constant strain amplitude strain ratios, *R*_*ε,OL*_ and *R*_*ε,CA*_, the overload and constant strain amplitude frequency, *f*_*OL*_ and *f*_*CA*_, the overload and constant strain amplitudes, *ε*_*a,OL*_ and *ε*_*a,CA*_, and the number of blocks to failure, *n*_*B*_.

**Table 2:** Summary of periodic overloading (PO) fatigue tests for Ti–6Al–4V ELI.

**Specimen ID**	***R***_***ε, OL***_	***R***_***ε, CA***_	***f***_***OL***_	***f***_***CA***_	***ε***_***a, OL***_	***ε***_***a, CA***_	***n***_***B***_
			**Hz**	**Hz**	**mm/mm**	**mm/mm**	**block**
**Fully-reversed,*****R***_***ε***_=**-1**							
PO_0.010-0.006(1)	−1	−1	1.0	3.0	0.010	0.006	282
PO_0.010-0.006(2)	−1	−1	1.0	3.0	0.010	0.006	238
PO_0.010-0.005(1)	−1	−1	1.0	5.0	0.010	0.005	400
PO_0.010-0.005(2)	−1	−1	1.0	5.0	0.010	0.005	318
PO_0.008-0.006(1)	−1	−1	1.0	3.0	0.008	0.006	1024
PO_0.008-0.006(2)	−1	−1	1.0	3.0	0.008	0.006	1606
PO_0.008-0.006(3)	−1	−1	1.0	3.0	0.008	0.006	1207
							
**Mean strain,*****R***_***ε***_=**0**							
PO_0.006R0-0.006R-1(1)	0	−1	1.0	3.0	0.006	0.006	843
PO_0.006R0-0.006R-1(2)	0	−1	1.0	3.0	0.006	0.006	872

### 2.3 Variable amplitude (VA) loading

**Fig. 3:**
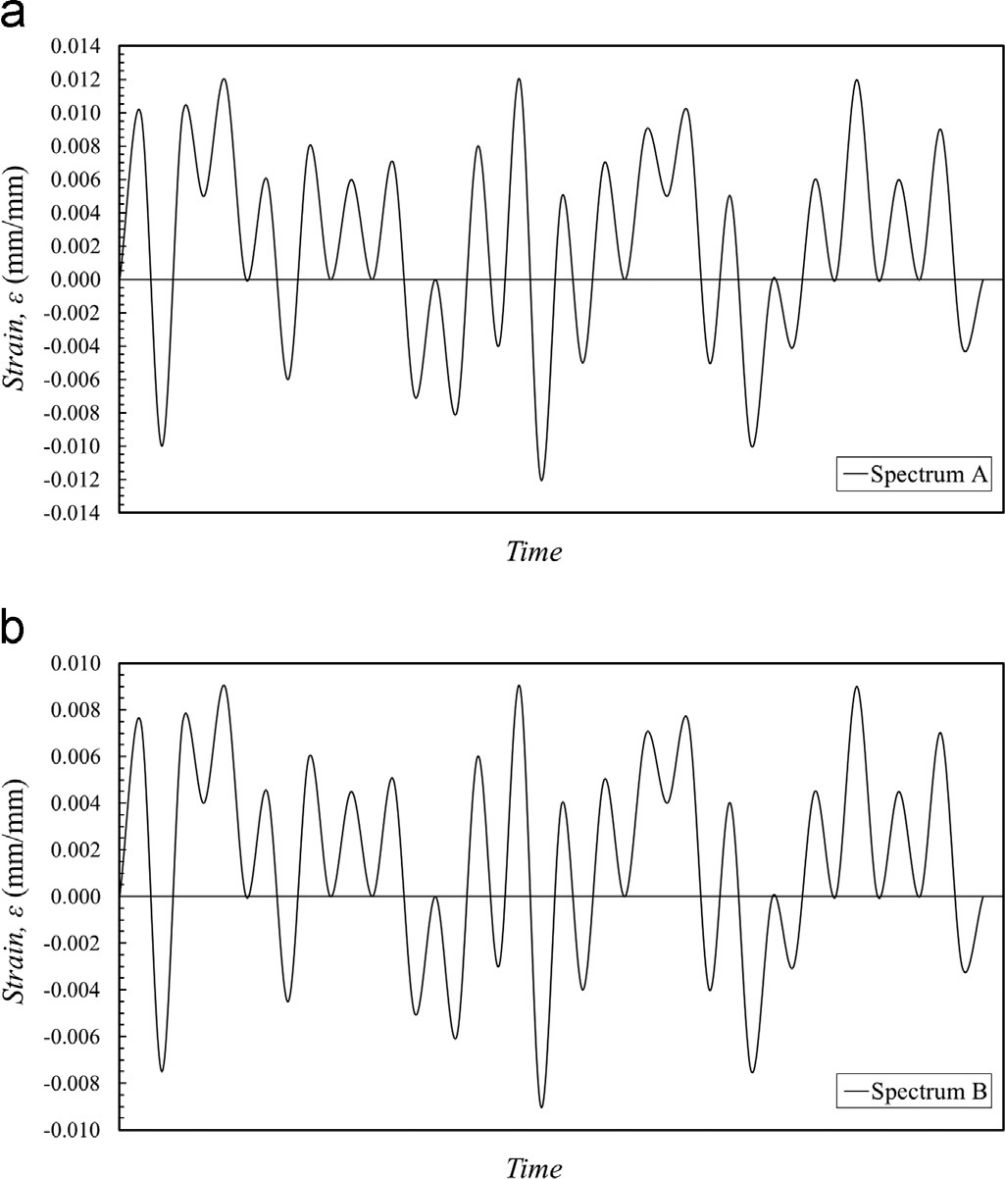
Loading spectrums used for variable amplitude (VA) tests, including (a) spectrum A and, (b) spectrum B.

Variable amplitude (VA) fatigue tests were conducted using a random strain loading spectrum. Fig. 3(a) illustrates the loading spectrum A, which consists of multiple cycles within the maximum and minimum strains of ±0.012 mm/mm. The loading block was repeatedly applied until the specimen reached failure. Fig. 3(b) shows spectrum B, which follows the same loading path as spectrum A, but within the maximum and minimum strain levels of ±0.009 mm/mm (75% reduction as compared to spectrum A). Table 3 lists the specimen ID, loading spectrum (A or B), and the number of blocks to failure, *n*_*B*_ for the strain-controlled VA fatigue tests.

**Table 3:** Summary of variable amplitude (VA) fatigue tests for Ti–6Al–4V ELI.

**Specimen ID**	***Loading spectrum***	***n***_***B***_
		**block**
VA_0.012_-0.012(1)	A	176
VA_0.012_-0.012(2)	A	172
VA_0.012_-0.012(3)	A	174
VA_0.009_-0.009(1)	B	470
VA_0.009_-0.009(1)	B	493

## 3 Disclaimer

The corresponding author of this article is on the editorial board of Data in Brief. The editorial and peer review process for this article was not handled by Nima Shamsaei. Furthermore, the authors of this article do not have access to any confidential information related to its peer-review process.
